# Highly Entangled, Mechanically Robust Hydrogel Thin Films for Passive Cooling Materials via Open-Vessel Fabrication

**DOI:** 10.3390/gels11090734

**Published:** 2025-09-12

**Authors:** Lihan Rong, Jiajiang Xie, Shigao Zhou, Tianqi Guan, Xinyi Fan, Wenjie Zhi, Rui Zhou, Feng Li, Yuyan Liu, Tingting Tang, Xiang Chen, Liyuan Zhang

**Affiliations:** 1 College of Physics and Electronic Information Engineering, Neijiang Normal University, Neijiang 641112, China; 2 Neijiang Optoelectronic Devices Engineering Research Center, Neijiang 641112, China; 3 College of Chemistry and Chemical Engineering, Neijiang Normal University, Neijiang 641112, China; 4 Key Laboratory of Fruit Waste Treatment and Resource Recycling of the Sichuan Provincial College, Neijiang 641112, China; 5 Special Agricultural Resources in Tuojiang River Basin Sharing and Service Platform of Sichuan Province, Neijiang 641112, China

**Keywords:** highly entangled gel, hydrogel, mechanical robust gel, passive cooling, PET-RAFT

## Abstract

The scalable fabrication of hydrogels with high toughness and low hysteresis is critically hindered by oxygen inhibition, which typically produces brittle, highly crosslinked (HC) networks. This study presents an oxygen-tolerant photoinduced electron transfer–reversible addition–fragmentation chain transfer (PET-RAFT) strategy for synthesizing highly entangled (HE) polyacrylamide hydrogels under open-vessel conditions. By optimizing the water-to-monomer ratio (W = 3.9) and introducing lithium chloride (LiCl) for spatial confinement, we achieved a fundamental shift in mechanical performance. The optimized HE hydrogel exhibited a fracture energy of 1.39 MJ/m^3^ and a fracture strain of ~900%, starkly contrasting the brittle failure of the HC control (W = 20, C = 10^−2^) at ~50% strain. This represents an order-of-magnitude improvement in deformability. Furthermore, the incorporation of 15 wt% LiCl amplified the HE hydrogel’s fracture energy to 2.17 MJ/m^3^ while maintaining its low hysteresis. This method enables the rapid, scalable production of robust, transparent thin films that exhibit dual passive cooling via radiative emission (>89% emissivity) and evaporation, rapid self-healing, and reliable strain sensing at temperatures as low as −20 °C. The synergy of entanglement design and confinement engineering establishes a versatile platform for manufacturing multifunctional hydrogels that vastly outperform their crosslink-dominated predecessors.

## 1. Introduction

Hydrogels have demonstrated tremendous potential across various fields. Among them, high-toughness hydrogels garner significant attention in the fields of wearable electronics [1,2,3], soft robotics [4,5,6], tissue engineering [7,8,9], wound dressing [10,11,12], and solar control [13,14,15]. The toughness of these hydrogels generally arises from the synergistic effects of elastic energy storage and viscous energy dissipation. Conventional toughening strategies predominantly focus on enhancing the energy dissipation capacity. For instance, double-network hydrogels utilize sacrificial bonds within highly crosslinked polymer chains to dissipate applied energy, together with methods to improve the mobility of interpenetrating networks. However, the viscoelastic behavior of polymer chains imposes inherent limitations: excessive energy dissipation often hampers recoverability, leading to permanent deformation, significant hysteresis effects, and diminished recovery rates. Those drawbacks compromise the stability and reliability in demanding applications, such as sensing and soft robotics.

Recent advancements propose an alternative paradigm—improving toughness through enhanced elasticity rather than amplified energy dissipation. Highly entangled hydrogels have emerged as promising platforms, where physical chain entanglements function as dynamic crosslinks. This architecture enables simultaneous tensile stiffening through chain orientation and energy storage via entropy-driven elasticity, thereby effectively minimizing hysteresis. Zhu et al. [16] developed a highly entangled double-network (HEDN) hydrogel, achieving a remarkable fracture energy (8340 J·m^−2^) and high tensile strength (up to 3 MPa), accompanied by extremely low hysteresis. Similarly, Chisa and colleagues [17] demonstrated a tough single-network polyacrylamide hydrogel exhibiting toughness up to 1.6 MJ·m^−3^. Nian et al. [18] used high-molecular-weight PEGDA chains for kneading and annealing to induce dense entanglements, followed by sparse crosslinking. This “dough-based” method successfully produced a hydrogel system with outstanding elasticity and negligible hysteresis effects.

Despite these successes, hydrogel fabrication remains constrained by oxygen-sensitive free-radical polymerization [19,20]. Due to the nature of free-radical polymerization (FRP), radicals are easily quenched by dissolved oxygen, terminating polymer chain growth. However, the degassing process is time consuming and costly, typically requiring an inert gas supply and a glove box or specialized apparatus—particularly critical when preparing thin hydrogel films where interfacial oxygen dominates inhibition. Beyond the above-mentioned limitation, another challenge involves the controllability of polymer chain growth inherent to commonly used ultraviolet (UV)-initiated polymerization. According to the Lake–Thomas model, fracture energy corresponds to the chain length between crosslinks. However, UV initiation inevitably induces side reactions and causes premature termination, restricting molecular weight and hindering the formation of ultra-high-molecular-weight (UHMW) polymer networks, therefore prohibiting the formation of a large number of entanglements. Consequently, these issues impose significant scalability obstacle for industrial production of highly entangled polymer hydrogels.

Photoinduced electron/energy transfer–reversible addition–fragmentation chain transfer (PET-RAFT) polymerization has been widely used to synthesize polymers under open-vessel conditions [21,22,23]. PET-RAFT polymerization typically employs a low concentration of a photoredox catalyst (PC) and utilizes visible light to initiate RAFT polymerization. Upon excitation, PCs are reduced to form radicals and anions, whose energy and electrons transfer to the thiocarbonylthio RAFT chain transfer agent and initiate polymerization [24]. In the presence of electron donors (such as tertiary amines, dimethyl sulfoxide, and ascorbic acid), the PCs mediate the conversion of triplet active oxygen into inert superoxide, conferring the oxygen-tolerance property [25,26]. Recent interest in PET-RAFT for hydrogel preparation stems from its aqueous solution compatibility, operation simplicity, and expandable chain-growth controllability [27,28]. Liu et al. [29] reported a bilayer hydrogel for wound dressing application with tunable inner- and outer-layer mechanical properties using PET-RAFT. However, the preparation takes over 12 h because of the low propagation rate of the active species. Chien et al. [30] reported a large-area hydrogel production method by using the PET-RAFT method. However, the compressive strength of the thin film was limited to lower than 20 kPa, which is insufficient for most coating applications. To date, the preparation of mechanically robust, highly entangled hydrogel thin films via PET-RAFT remains unreported.

In this study, we have demonstrated a straightforward method for preparing highly entangled (HE) polymer hydrogels under open-vessel conditions via PET-RAFT. The resulting HE hydrogel showed a remarkable work of fracture and low hysteresis effect. Introducing lithium chloride (LiCl) into the system accelerated polymerization, while spatial confinement concomitantly further enhanced the work of fracture, tensile strength, and Young’s modulus, and reduced hysteresis. These films enable passive cooling coatings by synergistically combining evaporative cooling and radiative cooling mechanisms. The LiCl-incorporated hydrogel also exhibited autonomous self-healing properties with trace amounts of water and functions as a strain sensor, establishing its potential for multifunctional thin film applications.

## 2. Results and Discussion

### 2.1. HE Hydrogel Preparation and Mechanical Properties

**Figure 1:**
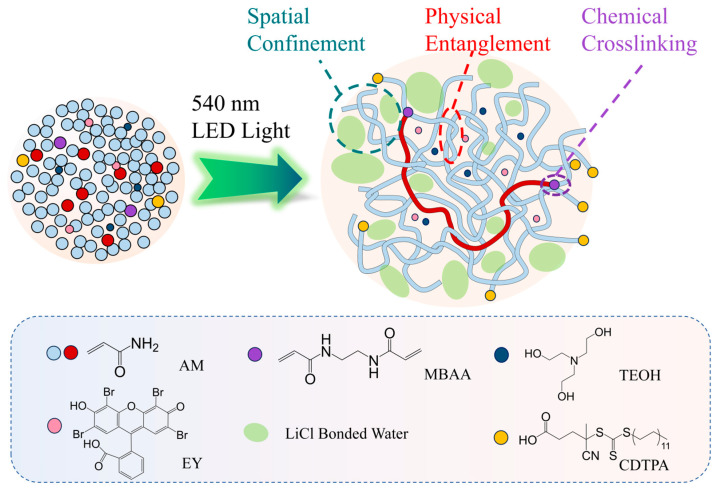
Schematic illustration of fabricating highly entangled hydrogel with PET-RAFT.

The preparation of the HE hydrogel utilized high-concentration monomer precursors. Herein, W denotes water-to-monomer molar ratio and C crosslinker-to-monomer molar ratio. When reducing W, the monomers were crowd distributed. During in situ polymerizing, chain propagation intrinsically facilitates entanglement formation. Unless otherwise specified, W = 3.9 was selected for HE hydrogel synthesis. The preparation process is shown in Figure 1.

Firstly, we prepared a high-concentration stock solution of Eosin Y (EY), 4-Cyano-4-(((dodecylthio)carbonothioyl)thio)pentanoic acid (CDTPA), and triethanolamine (TEOH). A desired amount of *N*,*N*′-methylenebisacrylamide (MBAA) was added to the solution, followed by dilution with deionized water. Subsequently, a specified quantity of acrylamide (AM) was added to the solution and vortex-mixed until complete dissolution. Finally, the solution was irradiated via 530 nm Light-Emitting Diode (LED) light with a custom apparatus, as shown in Figure S1. The final compositions of the different hydrogel samples are shown in Table 1. As shown in Figure S2, without crosslinkers (C = 0), and with W = 20, the samples failed to solidify after irradiation for 2 h. In contrast, the W = 3.9, C = 0 sample demonstrated successful solidification under identical irradiation conditions while keeping equivalent concentrations of EY, CDTPA, and TEOH.

**Figure 2:**
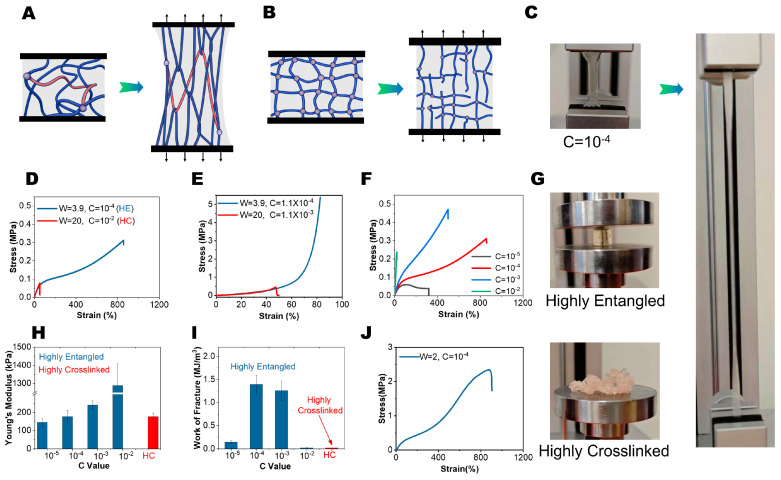
(**A**) Schematic illustration of stretching highly entangled (HE) hydrogel. (**B**) Schematic illustration of stretching highly crosslinked (HC) hydrogel. (**C**) Photograph of stretching HE hydrogel. (**D**) Tensile stress–strain curve for HE and HC hydrogel. (**E**) Compressive stress–strain curve for HE and HC hydrogels. (**F**) Tensile stress–strain curve for HE hydrogels with different C values. (**G**) Photograph of HE and HC hydrogels after compressive tests. (**H**) Young’s modulus of HE hydrogels with different C values and HC hydrogel. (**I**) Work of fracture of HE hydrogels with different C values and HC hydrogel. (**J**) Tensile stress–strain curve for HE hydrogels with W = 2.

HE hydrogels and highly crosslinked (HC) hydrogels exhibit different tensile behavior. As illustrated in Figure 2A, the elongated entangled chains undergo slippage and orient during the stretching process [16,31]. (The red color is to demonstrate a polymer strand to connect with another two polymer chains). This orientation redistributes the tensile stress through the adjacent long polymer chains, yielding ductile hydrogels without compromising stiffness. Conversely, HC networks localize stress concentration due to heterogenous crosslinking distribution and strand length variations, as shown in Figure 2B. Therefore, most of the HC hydrogels were stiff yet brittle materials. To compare the tensile properties of these systems, two samples were prepared with W = 3.9, C = 10^−4^ and W = 20, C = 10^−2^ to represent HE and HC hydrogels, respectively. Figure 2C demonstrates the extreme stretchability of HE hydrogels, which is further documented in Movie S1. Stress–strain curves (Figure 2D) revealed remarkable differences between HE and HC hydrogels: the HE hydrogel demonstrated ductile behavior, while the HC hydrogel fails brittlely. Even though Young’s modulus was similar, the HE hydrogel still demonstrated a higher tensile stress. The tensile-stiffening behavior observed in the HE samples at larger strains is attributed to the stress-induced orientation of polymer chains, as visually evidenced by specimen whitening, which is consistent with a previous publication [32]. Similar trends were corroborated in compression testing. As shown in Figure 2E, the HE sample withstood over 80% strain and recovered with minimum shape deformation, whereas the HC sample collapsed at 50% strain. Post-compression specimens further highlighted this distinctive mechanical response, as shown in Figure 2G.

To evaluate the influence of crosslinkers on mechanical properties, three additional samples were fabricated (C = 10^−5^, C = 10^−3^, and C = 10^−2^) to compare with the C = 10^−4^ sample. As shown in Figure 2F, the C = 10^−5^ hydrogel showed ductile tensile behavior, with a tensile strength of 0.05 MPa and strain at break of 300%. Increasing the ratio of crosslinkers to C = 10^−4^ elevated the tensile strength to 0.3 MPa and the strain at break to 900%. The above results demonstrated that the chemical crosslinks were necessary to prevent the chain from permanent dis-entanglements. To verify this, we tested the tensile behavior of the W = 3.9, C = 0 sample (Figure S3). In the low-strain region, the tensile stress increased linearly as expected, and demonstrated a high Young’s modulus of 184 kPa. When the strain reached 150%, the stress quickly decreased as the elongation increased. This could be due to the dis-entanglement of the long polymer chains. Instead, the sparsely distributed crosslinks prevented reptation of the polymer chains and thereby fixed the chain strand termini between junction points [33,34]. Further increasing the C value to C = 10^−3^ augmented the tensile strength to 0.5 MPa, but reduced the strain at break to 500%. At C = 10^−2^, the HE hydrogel transitioned to brittle behavior, in which the strain at break was greatly decreased to 50%. These results verified that chemical crosslinks restrict the dis-entanglement of the polymer chain and then enhance the stiffness, whereas excessive chemical crosslinks induce stress concentration and result in brittleness. Young’s moduli of the hydrogels are summarized in Figure 2H: with the increase in C value from 10^−5^ to 10^−3^, Young’s modulus increased from 145 kPa to 241 kPa, while the C = 10^−2^ sample showed a much higher Young’s modulus of 1289 kPa. The work of fracture is also summarized in Figure 2I: the results showed that the C = 10^−4^ sample showed the highest work of fracture of 1.39 MJ/m^3^, indicating that high entanglement could enhance stiffness with sacrificing toughness, contrasting sharply with HC hydrogels. The hydrogel exhibited exceptional strength, capable of carrying 1.5 kg of water masses, as shown in Figure S4.

Further reducing the W value significantly enhanced the mechanical properties. As shown in Figure 2J, when reducing W to 2 while keeping C = 10^−4^, the hydrogel showed an excellent tensile strength of 2.5 MPa and retained a fracture strain of 900%. Young’s modulus also increased to 465 kPa, indicating substantial stiffening through high entanglement density. Comparing these results to recent publications on highly entangled systems, our results demonstrate a substantial enhancement in tensile strength and strain at break, exceeding the values reported for similar systems (0.12~0.39 MPa and 300~570% of the literature value, as shown in Table 2). We attribute this improvement to the unique characteristics of the PET-RAFT polymerization technique. We posit that, by minimizing chain termination events, PET-RAFT fosters a more uniform polymer architecture. This likely reduces the population of short chains and mitigates inhomogeneous network distributions that typically form during conventional synthesis [35]. Further increasing W to 8 produced a solid hydrogel exhibiting insufficient rigidity for mechanical characterization.

### 2.2. HE Hydrogel with Spatial Confinement

**Figure 3:**
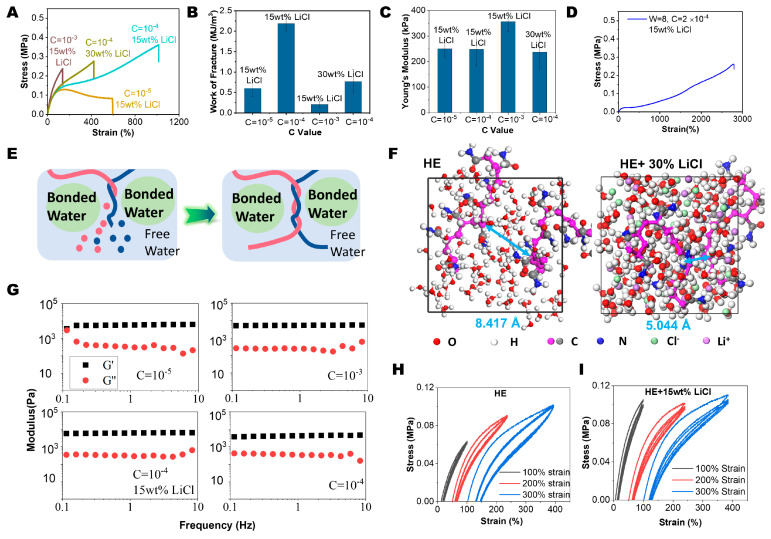
(**A**) Tensile stress–strain curve for HE hydrogel with LiCl spatial confinement. (**B**) Work of fracture for HE hydrogel with LiCl spatial confinement. (**C**) Young’s modulus for HE hydrogel with LiCl spatial confinement. (**D**) Tensile stress–strain curve for HE hydrogel with W = 8 and 15 wt% LiCl. (**E**) Schematic illustration of possible mechanism of strengthening and solidification boosting.(Colors represnted different chains) (**F**) Molecular dynamics simulation for the distance between two PAM chains under pure water/water+LiCl environments.(The arrow is to show the smallest distances) (**G**) G′ and G″ for HE hydrogels with different C values and LiCl confinement. (**H**) Load–unload cycles for HE hydrogel and (**I**) HE hydrogel with LiCl spatial confinement under 100, 200, and 300% strain.

**Figure 4:**
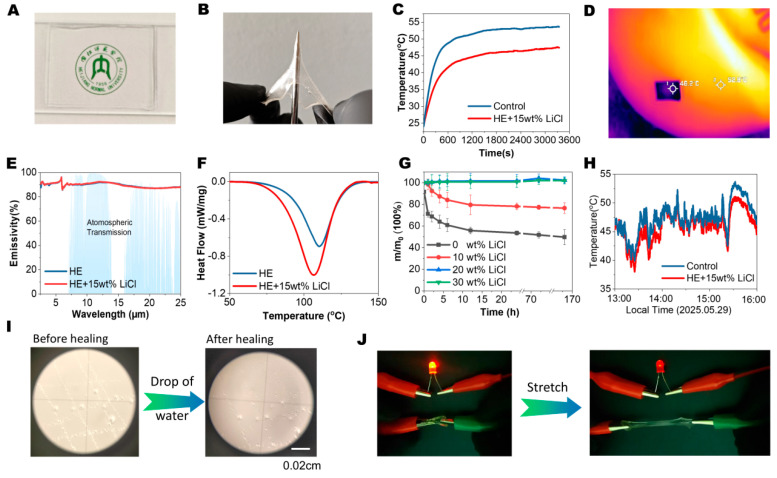
(**A**) HE-LiCl hydrogel thin film showing high transparency. (**B**) HE-LiCl hydrogel thin film showing puncturing resistance. (**C**) Passive cooling property of the HE-LiCl hydrogel thin film for indoor simulation test. (**D**) IR image showing the passive cooling property of HE-LiCl hydrogel thin film for outdoor passive cooling capacity. (**E**) Emissivity of the HE and HE-LiCl hydrogel thin films under long wavelength range and comparison with atmospheric transmission. (**F**) DSC study of the evaporating enthalpy for HE and HE-LiCl thin films. (**G**) Moisture retention of HE hydrogel thin films with different loading of LiCl. (**H**) Passive cooling property of the HE-LiCl hydrogel thin film in actual outdoor tests. (**I**) Microscope image demonstrating the scratch-healing properties of HE-LiCl. (**J**) Images of HE-LiCl thin films for strain sensor applications.

Introducing spatial confinement to polymer chains could greatly enhance mechanical properties by restricting polymer chain movements. For instance, Wang et al. [38] used CaCl_2_ to prepare low-hysteresis hydrogels by polymerizing the hydrogels in a salt solution, where the Ca^2+^-bonded water constrained the polymer chains within the free water regions and enhanced the fracture- and fatigue-resistant properties of the hydrogels. In this study, LiCl was selected to create spatial confinements due to its strong water affinity. The results shown in Figure 3A demonstrated that 15 wt% (LiCl to water) of LiCl could increase the tensile stress of the C = 10^−4^ hydrogel to 0.4 MPa while maintaining the strain at break at 900%, while the C = 10^−5^ sample showed similar results (tensile strength increased from 0.05 MPa to 0.15 MPa while keeping the strain at break at 500%), showing mechanical enhancement via LiCl. The work of fracture is summarized in Figure 3B, in which the WOF of the C = 10^−5^ and C = 10^−4^ hydrogels increased to 0.60 and 2.17 MJ/m^3^, respectively, demonstrating the increases in toughness. Young’s modulus of the C = 10^−4^ hydrogel with 15 wt% LiCl increased to 356 kPa, while the other samples showed similar Young’s moduli at around 250 kPa, as depicted in Figure 3C, indicating an improvement in stiffness. Moreover, the tensile strength of the hydrogel also improved to 0.15 MPa, when compared with that of 0.05 MPa for samples without LiCl. Notably, as shown in Figure 2D, the W = 8 and C = 2 × 10^−4^ sample could be synthesized with 15 wt% of LiCl, achieving a good tensile strength of 0.3 MPa and extraordinary strain at break of 2800%, demonstrating that spatial confinement also helps the formation of hydrogels, even with lower concentrations of monomers. The mechanism of enhancement could be as follows: polyacrylamide (PAM) has relatively lower hydration-free energy than ions, which led water to migrate and form bonded water regions, thus forcing the PAM chain to come together, as shown in Figure 4E and Scheme S1 [38]. Therefore, those spatial confinement regions would act as additional physical crosslinks and therefore enhance the hydrogel’s mechanical properties. Molecular dynamics simulation confirmed this hypothesis by calculating the distance between PAM oligomers. As shown in Figure 4F, the optimized configuration showed that the distance between the PAM chains in the pure water system was 8.417 Å, while that in the system with 30 wt% LiCl was 5.044 Å, supporting the squeezing of PAM chains with bonded water regions. However, the C = 10^−3^ samples showed a compromised work of fracture, tensile strength, and strain at break. We attribute this to excessive confinement. As illustrated in Figure 3E, the bonded water severely limited the free volume for chain movement, obstructing the chain slip mechanism essential for ductility. Furthermore, while the high monomer concentration promoted entanglement, an overabundance of these physical crosslinks ultimately restricted chain mobility. Therefore, too much spatial confinement along with entanglement could embrittle the hydrogel, similarly to chemical crosslinks. Rheology measurements shown in Figure 3G indicated that, under the measurement conditions, the storage modulus (G′) was higher than the loss modulus (G″), indicating a stable elastic solid behavior. Furthermore, adding LiCl enhanced the resilience of the hydrogel. Load–unload cycling was performed at 100%, 200%, and 300% strain at a fast deformation rate of 100 mm/min, as shown in Figure 2I and Figure 3H. The results showed that under 100% strain, the energy dissipation was nearly perfect, with both samples showing high elasticity. In the 200% and 300% strain cycles, the LiCl-added sample showed smaller hysteresis loops, indicating lower energy dissipation and faster recovery properties. After the first cycle, the second and third cycles showed negligible difference, confirming exceptional elastic resilience.

Notably, incorporation of LiCl into the polymerization step accelerated the solidification rate under open-vessel conditions. As shown in Figure S5, after exposure to LED light for 30 min, the sample without LiCl was viscosified but remained fluid, while the sample with added LiCl had fully solidified. The precursor solution with LiCl also cured under natural sunlight. As shown in Figure S6, after solar exposure for 8 h, the sample cured spontaneously, whereas when putting them into a dark environment, no sol–gel transition was observed. In contrast, the sample without LiCl remained liquid under daylight even after over 48 h of daylight exposure. The possible mechanism of the speed-up phenomenon could also be explained via spatial confinements. As shown in Figure 2E, the bonded water regions would repel the PAM chains and squeeze them together. Therefore, the free water region between bonded water regions would have an effectively increased monomer concentration, which would accelerate the polymerization kinetics. Concurrently, polymerization within confined spaces promotes entanglement formation that stabilizes the hydrogel network architecture.

### 2.3. Passive Cooling Thin Film and Strain Sensor Applications

This simple preparation method demonstrated remarkable potential in fabricating multifunctional thin films. Conventional hydrogel thin film production involves a degassing process to eliminate the dissolved oxygen that inhibits polymerization. Oxygen inhibition is particularly pronounced in thin films due to their high surface-to-volume ratio, compromising quality and impeding scalable manufacturing. In this study, we have utilized the PET-RAFT polymerization method, which has the property of oxygen tolerance. As shown in Scheme S2, firstly, the precursor solution was deposited on a glass slide. Then spacers with defined thickness (1 mm) were placed on the glass slide. Then a polyethylene terephthalate (PET) film was placed on the surface to reduce the direct contact between air and the solution. Then the thin film was irradiated under LED light until the thin film formed (typically cured with 10 min). During the polymerization, the color of the hydrogel film changed from orange to faint yellow due to the decomposition of EY. The resultant highly transparent film is shown in Figure 4A. Remarkably, these films exhibited outstanding puncture resistance, as shown in Figure 3B. Under substantial indentation displacement, no crack propagation originated from the puncture site.

These robust and transparent thin films are promising candidates for passive cooling materials. Passive cooling materials are engineered surfaces designed to lower the temperature without an external energy supply, offering energy efficiency and environmental applications. Transparency is essential in some scenarios, such as solar cell applications. We firstly evaluated the passive cooling performance indoors under infrared light to simulate a solar source. The schematic diagram of the experiments is shown in Scheme S3. In brief, the thin film was placed on a glass slide which was on top of a heat-insulating form, and the temperature below the hydrogel was measured with thermocouples. The results showed that, upon irradiation, hydrogel-film-coated sections exhibited slower temperature elevation rates and lower equilibrium temperatures versus the control, showing around 6 °C of temperature reduction. The infrared camera image showed consistent results between the simulation measurements and those under actual daylight, in which the temperature difference between the film-coated and bare surfaces was around 6.6 °C, as shown in Figure 4D. The cooling properties originate from two mechanisms: radiative cooling and evaporative cooling. As shown in Figure 4E, the HE hydrogel showed a high emissivity (>89%) in the range of 7–14 μm (the atmosphere window) both with and without LiCl. In this region, the atmosphere’s low absorptivity allows the hydrogel’s emissivity to radiate directly into outer space, effectively dissipating heat. On the other hand, the evaporation of the hydrogel could also contribute to the cooling properties, especially under low-humidity conditions. Figure 4F shows the differential scanning calorimetry (DSC) curve of the samples with and without LiCl. The larger peak of HE-LiCl hydrogel at around 105 °C showed that the evaporating enthalpy was increased via adding LiCl, implying a potential increase in cooling properties. This higher evaporating enthalpy was also detected in the mass retaining tests. As shown in Figure 4G, thin films without LiCl demonstrated a fast evaporating rate when stored under room-temperature and open conditions. Via adding 10 wt% of LiCl, the mass change in the hydrogel was largely reduced. When increasing the concentration of LiCl to 20 and 30%, the hydrogel could maintain its original mass after storage for 7 days, showing its capability for long-term storage and usage. We also performed field tests under actual daylight in Neijiang, China (29°36′57″ N, 105°6′7″ E). In this study, we placed the setup shown in Scheme S3 on a rooftop and recorded the temperature difference between the control group and coated group. The results are shown in Figure 4H, indicating an overall temperature reduction in the period of 9:00–15:00 PM.

Furthermore, these thin films also showed potential self-healing capability. As shown in Figure 4I, the thin film was scratched with a knife. Then a drop of water was added to the surface. After 2 min, the scratch disappeared, showing a rapid self-healing advantageous for wearing-resistance applications. Figure 4J showcases the strain sensor functionality. When connected into a closed circuit, the hydrogel controlled the brightness of the LED light bulb via stretching. The stretching process modulated the resistance of the hydrogel and thus influenced the brightness of the light bulb. Moreover, because of the high concentration of LiCl, the hydrogel also demonstrated low-temperature tolerance. After storage in a refrigerator (−20 °C) for over 24 h, the hydrogel still remained flexible and bendable, as shown in Movie S3, indicating the potential for fabricating electronic devices for low-temperature applications. Critically, strain-sensing functionality persisted at −20 °C (Figure S7), maintaining the stretch-induced resistance variation essential for cryogenic applications.

## 3. Conclusions

In conclusion, we have developed a simple open-vessel strategy to synthesize robust, multifunctional hydrogels through the synergistic combination of chain entanglement and ionic spatial confinement. By minimizing the water-to-monomer ratio (W ≤ 3.9), we fostered the formation of a highly entangled network architecture, an alternative to traditional highly crosslinked designs. This approach yielded hydrogels with an exceptional combination of high toughness (up to 2.17 MJ/m^3^ with LiCl), extreme stretchability (~900%), and low hysteresis.

Spatial confinement via LiCl played a dual role, simultaneously enhancing mechanical properties and accelerating polymerization under ambient conditions. The oxygen-tolerant PET-RAFT method enabled the scalable fabrication of high-quality, transparent thin films without degassing.

These robust films demonstrated multifunctionality, serving as effective passive cooling materials via radiative and evaporative mechanisms, rapid self-healing coatings, and reliable strain sensors operable even at −20 °C. This work establishes a versatile and industrially viable platform for manufacturing advanced hydrogels for applications in sustainable cooling, wearable electronics, and adaptive coatings.

## 4. Materials and Methods

### 4.1. Materials

Acrylamide (AM), *N*,*N*′-methylenebisacrylamide (MBAA), Eosin Y (EY), Triethanolamine (TEOH) 4-Cyano-4-(((dodecylthio)carbonothioyl)thio)pentanoic acid (CDTPA), and lithium chloride (LiCl) were purchased from Shanghai Aladdin Biochemical Technology Co., Ltd. (Shanghai, China).

### 4.2. Synthesis of Poly(acrylamide) (PAM) HE Hydrogels

The synthesis procedure was conducted according to a previous report by our group [39]. EY and TEOH were dissolved in deionized water with concentrations of 1 mg/mL and 4 mg/mL, respectively, to prepare stock solution 1. In total, 1 mL of 5 mg/mL CDTPA in DMSO solution and 9 mL of stock solution 1 were added to a 20 mL vial. A predetermined quantity of MBAA was added to stock solution 1 and dissolved by heating in a water bath to prepare stock solution 2. A total of 0.5 mL of stock solution 2, a calculated amount of water (by W), a predetermined amount of AM (by C), and the desired amount of LiCl was added to the solution and mixed until the solids were totally dissolved. The resulting precursor solution was poured into a dogbone-like PTFE mold for further tensile testing and a cylinder silicone rubber mold for compressive testing. Then the molds were exposed to an LED light (530 nm) for the desired time, with a light intensity of 2.79 mW/cm^2^.

### 4.3. Fabrication of HE Hydrogel Thin Film

Fabrication of HE hydrogel thin films utilized the above-mentioned precursor solution (see Supporting Information for scheme). Two spacers were placed on a glass slide to adjust the film thickness to 1 mm. Then the precursor solution was drop-casted on a glass slide between the spacers. Then a PET cover was placed on the spacer to flatten the liquid surface. Then the thin films were placed under LED light (540 nm; 2.79 mW/cm^2^) (Xuzhou Aijia Co., Xuzhou, China) until the liquid solidified. Then the PET cover was removed and the thin film was peeled off from the glass slide for further study.

### 4.4. Characterization

#### 4.4.1. Mechanical Tests

The mechanical property testing was performed by using an electronic universal test machine (Dongguan Lixian Experimental Instrument, Dongguan, China). The tensile testing mold and compressive testing mold were purchased from Haihong Precision Mold Co. (Liaocheng, China), with a dogbone shape of gauge dimension of 50 mm × 8.5 mm × 2 mm and a cylindrical shape of 10 mm diameter and 10 mm in height, respectively. The tensile and compressive speeds were set as 100 mm/min unless stated otherwise. The work of fracture of the hydrogels was determined from the area under the tensile stress–strain curve. Young’s modulus was calculated from the slope of the linear region of the tensile stress–strain curve. The load–unload cycle was performed with a load/unload speed of 100 mm/min with desired strain. The rheological properties were tested using a MARS 60 rheometer (Thermo Fisher, Waltham, MA, USA). The tests were performed using a cylinder of 10 mm diameter and 10 mm heights, with a frequency sweep from 0.1 to 10 Hz under 1% strain.

#### 4.4.2. Passive Cooling Tests

The passive cooling tests were performed with a test device made with an insulating form wrapped with aluminum foil (see Supporting Information) to eliminate heat transfer. The sample was placed on a glass slide and then placed on the top of the test device. Another control group was tested at the same time with a glass slide only. Then the values of temperature were measured by K-type thermocouples (ART Technology, Shenzhen, China) and recorded with an ART Technology DAM-3134 data acquisition module (ART Technology, Shenzhen, China). For the indoor simulation tests, an infrared (IR) light (OAR38 IR 100R) (Philips (China), Shanghai, China) was used as the simulated light source. The test device was placed under the IR light at a distance that controlled the irradiance on the sample surface to be 20 mW/cm^2^. For the actual outdoor test, the samples were placed on a rooftop in Neijiang, Sichuan, China (29°36′57″ N, 105°6′7″ E) on 28 May 2025, and the data was recorded with the same test device. The IR image was recorded on the same date with a Flir One Pro IR camera(Teledyne FLIR LLC, Houston, TX, USA). The emissivity (ε) was calculated via transmissivity (τ) and reflectivity (ρ) by ε = 1 − τ − ρ, where τ and ρ were measured by a Nicolet IS550 FTIR (Thermo Fisher, Waltham, MA, USA) spectrometer equipped with a gold integrating sphere. Differential scanning calorimetry (DSC) curves were measured with a NETZSCH DSC 200 F3 (NETZSCH, Selb, Germany) with a heating rate of 10 °C/min from room temperature to 150 °C.

#### 4.4.3. Self-Healing Observation and Strain Sensor Tests

The self-healing observation was performed on an as-prepared thin HE hydrogel film. The thin film surface was scratched with a knife, and a drop of water was added on the scratched surface and left to stand for 2 min. The self-healing process was observed with a microscope (Changchun Great Wall Teaching Instrument, Changchun, China). The potential strain sensor test was performed using a Fluke 17B Digital Multimeter (Fluke, Everett, WA, USA) as a power supply.

#### 4.4.4. Molecular Dynamics Simulation

Molecular dynamics (MD) simulations were performed using BIOVIA Materials Studio 2023 with a COMPASS III forcefield and forcefield-allocated partial charges. PAM oligomers with eight repeating units were solvated in periodic boxes constructed via the Amorphous Cell module: (1) an aqueous system (two PAM chains in 80 water molecules) and (2) a 30 wt% LiCl solution (H_2_O:Li^+^:Cl = 5:1:1). NVT ensemble simulations at 297.15 K (Nosé–Hoover thermostat) were conducted for 300 ns using the Forcite module.

## Figures and Tables

**Table 1:** Final compositions of hydrogel samples.

Sample	W Value	C Value	LiCl (wt%)	AM (g)	MBAA (mg)	EY (mg)	TEOH (mg)	CDTPA (mg)	Water (mL)	LiCl (g)
HE	3.9	0	0	1.0 ± 0.1	0	0.025 ± 0.002	2.0 ± 0.1	0.25 ± 0.02	1.0 ± 0.1	0
HC	20	0	0	0.20 ± 0.02	0	0.025 ± 0.002	2.0 ± 0.1	0.25 ± 0.02	1.0 ± 0.1	0
HE	3.9	10^−5^	0	1 ± 0.1	0.0025 ± 0.0002	0.025 ± 0.002	2.0 ± 0.1	0.25 ± 0.02	1.0 ± 0.1	0
HE	3.9	10^−4^	0	1 ± 0.1	0.025 ± 0.002	0.025 ± 0.002	2.0 ± 0.1	0.25 ± 0.02	1.0 ± 0.1	0
HE	3.9	10^−3^	0	1 ± 0.1	0.25 ± 0.02	0.025 ± 0.002	2.0 ± 0.1	0.25 ± 0.02	1.0 ± 0.1	0
HE	3.9	10^−2^	0	1 ± 0.1	2.5 ± 0.2	0.025 ± 0.002	2.0 ± 0.1	0.25 ± 0.02	1.0 ± 0.1	0
HC	20	10^−2^	0	0.20 ± 0.02	2.5 ± 0.2	0.025 ± 0.002	2.0 ± 0.1	0.25 ± 0.02	1.0 ± 0.1	0
HE	2	10^−4^	0	2 ± 0.1	0.025 ± 0.002	0.025 ± 0.002	2.0 ± 0.1	0.25 ± 0.02	1.0 ± 0.1	0
HE-LiCl	3.9	10^−5^	15	1 ± 0.1	0.0025 ± 0.0002	0.025 ± 0.002	2.0 ± 0.1	0.25 ± 0.02	1.0 ± 0.1	0.15 ± 0.02
HE-LiCl	3.9	10^−4^	15	1 ± 0.1	0.025 ± 0.002	0.025 ± 0.002	2.0 ± 0.1	0.25 ± 0.02	1.0 ± 0.1	0.15 ± 0.02
HE-LiCl	3.9	10^−3^	15	1 ± 0.1	0.25 ± 0.02	0.025 ± 0.002	2.0 ± 0.1	0.25 ± 0.02	1.0 ± 0.1	0.15 ± 0.02
HE-LiCl	3.9	10^−3^	30	1 ± 0.1	0.25 ± 0.02	0.025 ± 0.002	2.0 ± 0.1	0.25 ± 0.02	1.0 ± 0.1	0.30 ± 0.04
HE-LiCl	8	2 × 10^−4^	15	0.50 ± 0.03	0.05 ± 0.04	0.025 ± 0.002	2.0 ± 0.1	0.25 ± 0.02	1.0 ± 0.1	0.15 ± 0.03

AM: Acrylamide; MBAA: *N*,*N*′-methylenebisacrylamide; EY: Eosin Y; TEOH: Triethanolamine; CDTPA: 4-Cyano-4-(((dodecylthio)carbonothioyl)thio)pentanoic acid.

**Table 2:** Comparison of tensile strength and tensile strain with recent publications.

Materials	Year	Tensile Strength (MPa)	Tensile Strain (%)
PAAm [31]	2021	0.39	~420
PAAm [17]	2021	~0.3	\
PMPC [17]	2021	~0.14	~330
Poly(methyl methacrylate) (PMMA) [36]	2022	0.12	570
PEG [18]	2022	0.46	590
PAAm [32]	2024	0.18	\
P(AAm-co-AMPS) [16]	2024	\	~320
poly(N-iso-propylacrylamide)+hydroxypropyl cellulose(PNIPAM+HPC) [37]	2023	0.05	500
This work		0.49~2.5	900

## Data Availability

The original contributions presented in this study are included in the article. Further inquiries can be directed to the corresponding authors.

## Supplementary Materials

The following supporting information can be downloaded at: https://www.mdpi.com/article/10.3390/gels11090734/s1, Figure S1. Apparatus of shining LED light for PET-RAFT polymerization. Figure S2. Photographs of W = 20, C = 0 and W = 3.9, C = 0 samples. Figure S3. Stress—Strain curve of W = 3.9, C = 0 sample under tensile test. Figure S4. HE hydrogel for carrying heavy objects. Scheme S1. Schematic illustration of spatial confinement. Figure S5. Photograph of hydrogel with/without LiCl after irradiating for 30 min. Figure S6. Sample curing under daylight. Scheme S2. Schematic illustration of preparation of HE hydrogel thin film. Scheme S3. Schematic illustration of passive cooling property measurements. Figure S7. Connecting HE-LiCl hydrogel at −20 °C into circuit. Movie S1: Highly Crosslinked Hydrogel. Movie S2: Highly Entangled Hydrogel. Movie S3: After storage in a refrigerator (−20 °C) for over 24 h, the hydrogel still remained flexible and bendable.
