# Supplementary material for: Highly Entangled, Mechanically Robust Hydrogel Thin Films for Passive Cooling Materials via Open-Vessel Fabrication
Source: Gels. 2025 Sep 12;11(9):734. doi: 10.3390/gels11090734 (PMC12469944; doi:10.3390/gels11090734)
Supplement: Supplementary file 1 [file gels-11-00734-s001.zip › gels-3858637-supplementary-File S1.pdf]

Supporting Information

Highly Entangled, Mechanically Robust Hydrogel Thin Films for Passive Cooling Materials Via Open-Vessel Fabrication

Li-Han Rong,[a b] **Jiajiang Xie,*[a] *Shigao Zhou,*[a] *Tianqi Guan,*[a] *Xinyi Fan,*[a] *Wenjie Zhi,*[a] *Rui Zhou,*[a] *Feng Li,*[a] *Yuyan Liu,*[a] *Tingting Tang,*[a] *Xiang Chen* [a], *Liyuan Zhang* [c,d,e]

[a] College of Physics and Electronic Information Engineering , Neijiang Normal University, Neijiang 641112, P.R. China

[b] Neijiang Optoelectronic Devices Engineering Research Center, Neijiang 641112, P.R. China

[c] College of Chemistry and Chemical Engineering, Neijiang Normal University, Neijiang， 641112, China;

[d] Key Laboratory of Fruit Waste Treatment and Resource Recycling of the Sichuan Provincial College, Neijiang， 641112, China;

[e] Special Agricultural Resources in Tuojiang River Basin Sharing and Service Platform of Sichuan Province, Neijiang， 641112, China

Corresponding Authors [*]: Li-Han Rong (rlihan@njtc.edu.cn)

Table of Content

Figure. S1. Apparatus of shining LED light for PET-RAFT polymerization. 3

Figure. S2. Photographs of W=20, C=0 and W=3.9, C=0 samples 4

Figure. S3. Stress- Strain curve of W=3.9, C=0 sample under tensile test 5

Figure. S4. HE hydrogel for carrying heavy objects. 6

Scheme S1. Schematic illustration of spatial confinement. 7

Figure. S5. Photograph of hydrogel with/without LiCl after irradiating for 30 mins.... 8

Figure. S6. Sample curing under daylight 9

Scheme S2.Schematic illustration of preparation of HE hydrogel thin film. 10

Scheme S3. Schematic illustration of passive cooling property measurements 11

Figure. S7. Connecting HE-LiCl hydrogel at -20 °C into circuit................................ 12

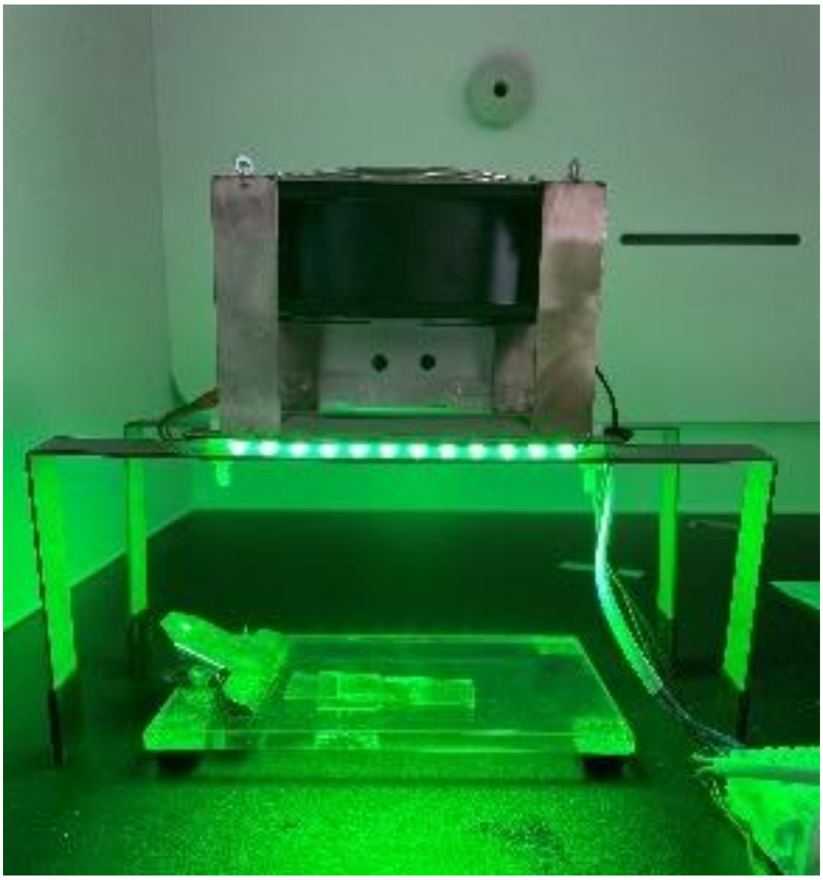

Figure. S1. Apparatus of shining LED light for PET-RAFT polymerization.

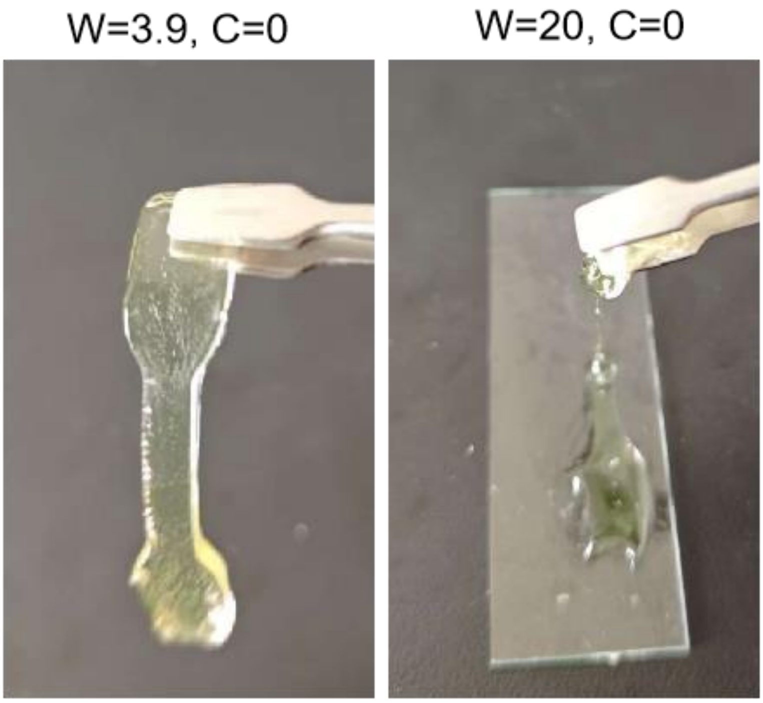

Figure. S2. Photographs of W=20, C=0 and W=3.9, C=0 samples

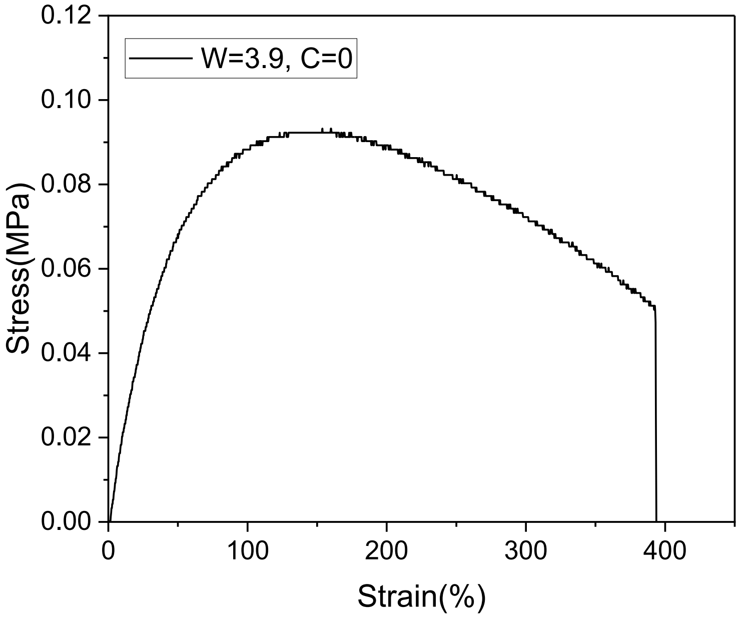

Figure. S3. Stress- Strain curve of W=3.9, C=0 sample under tensile test

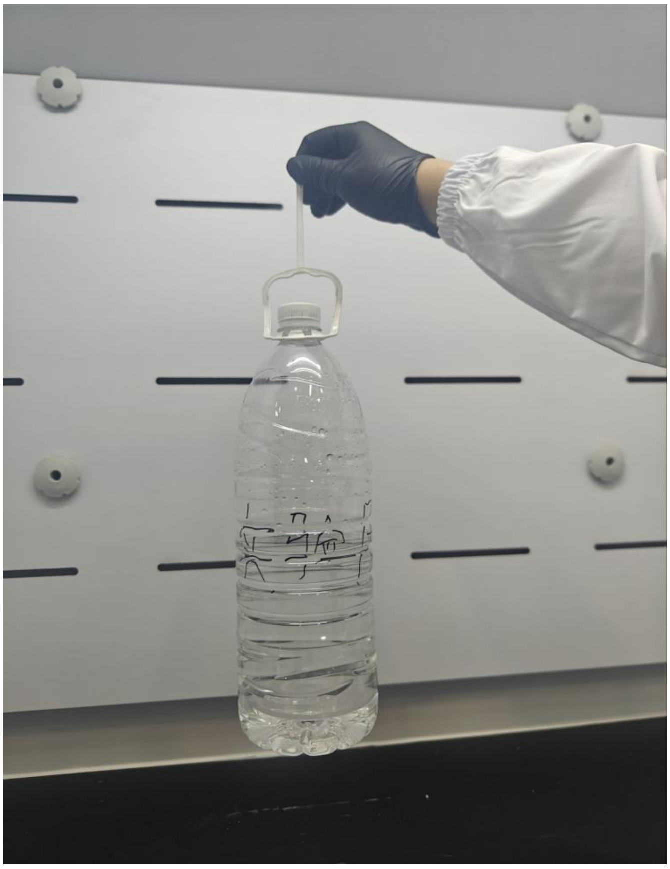

Figure. S4. HE hydrogel for carrying heavy objects.

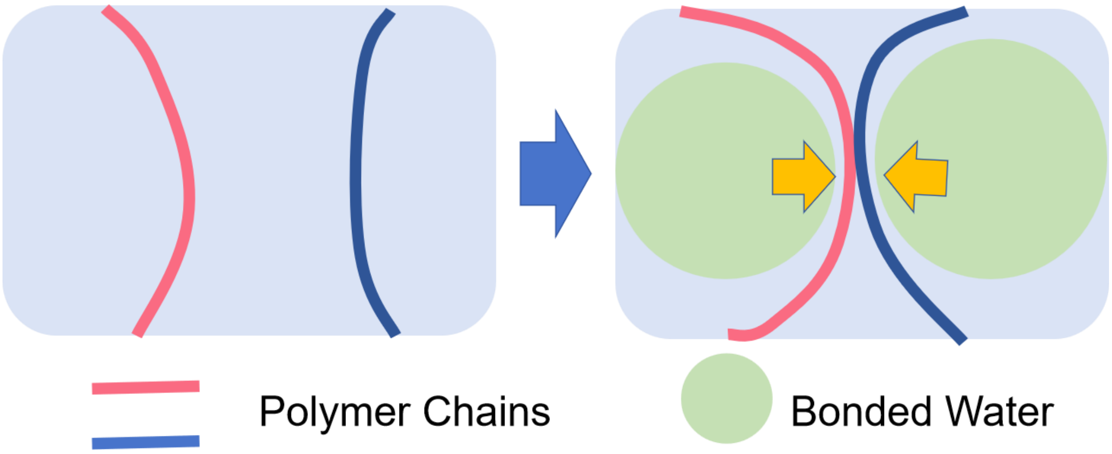

Scheme S1. Schematic illustration of spatial confinement.

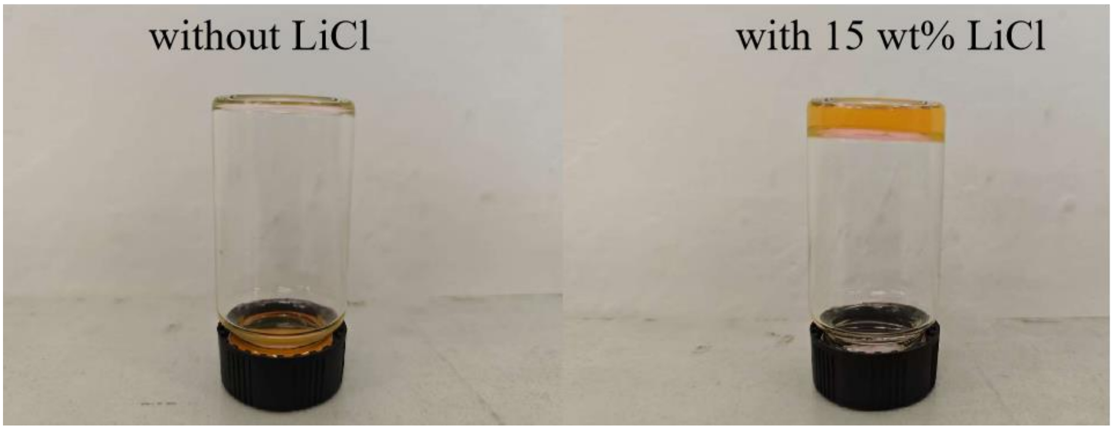

Figure. S5. Photograph of hydrogel with/without LiCl after irradiating for 30 mins

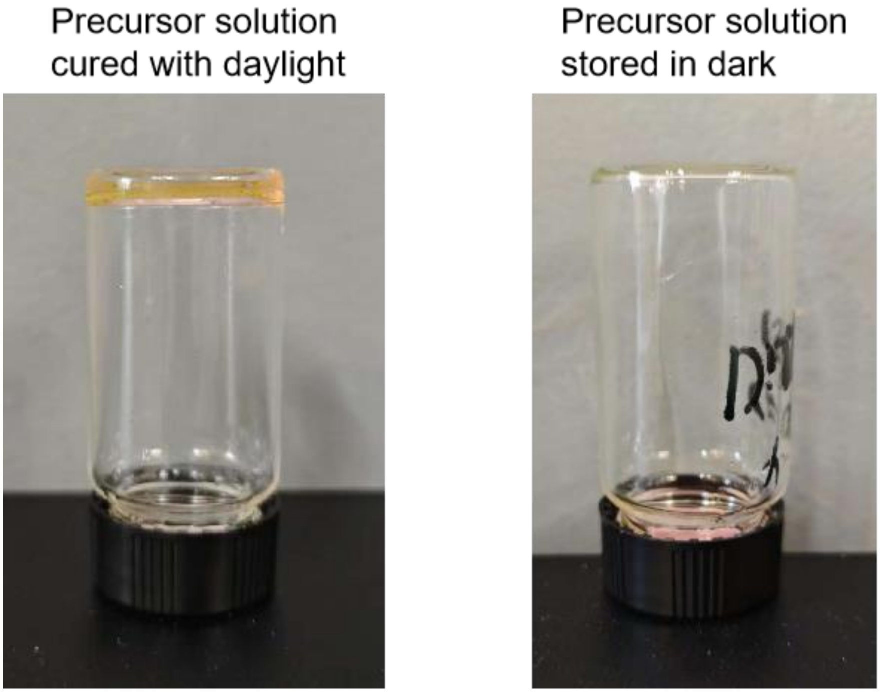

Figure. S6. Sample curing under daylight

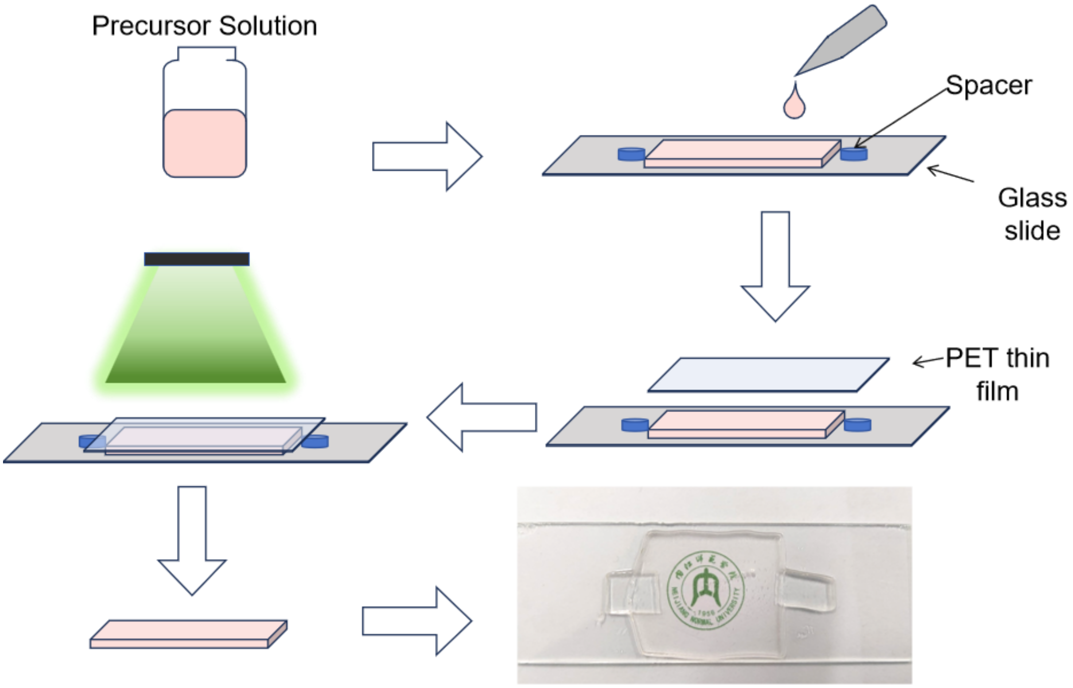

Scheme S2.Schematic illustration of preparation of HE hydrogel thin film.

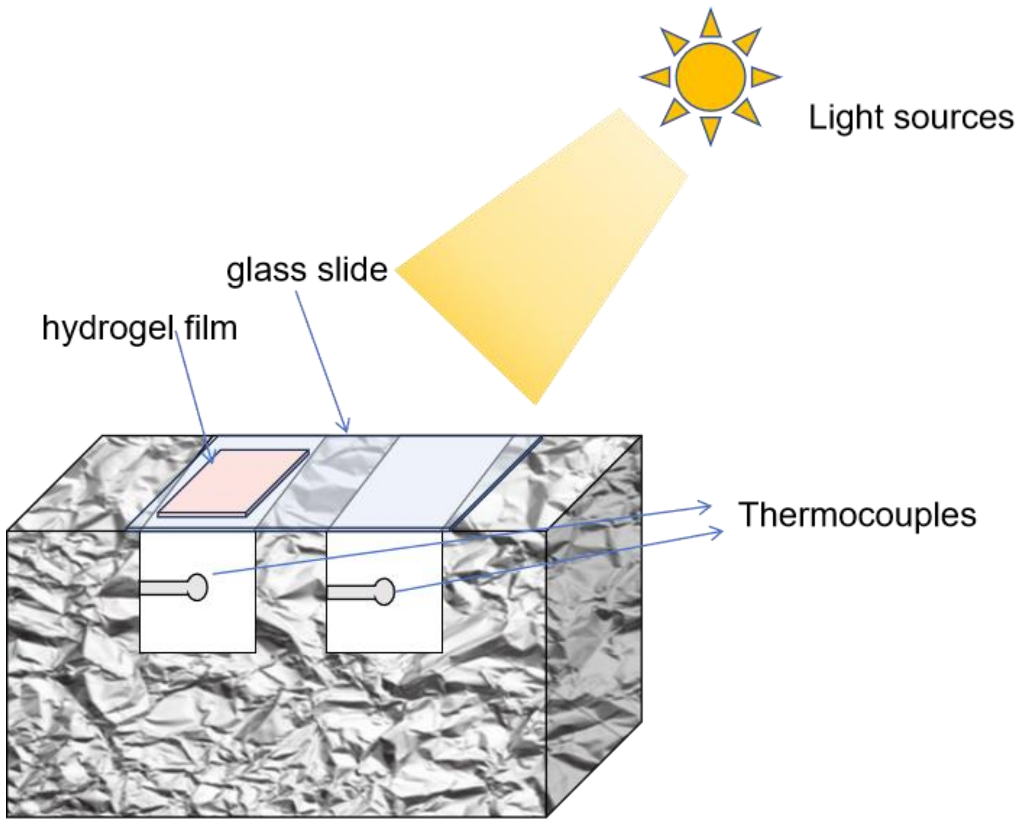

Scheme S3. Schematic illustration of passive cooling property measurements

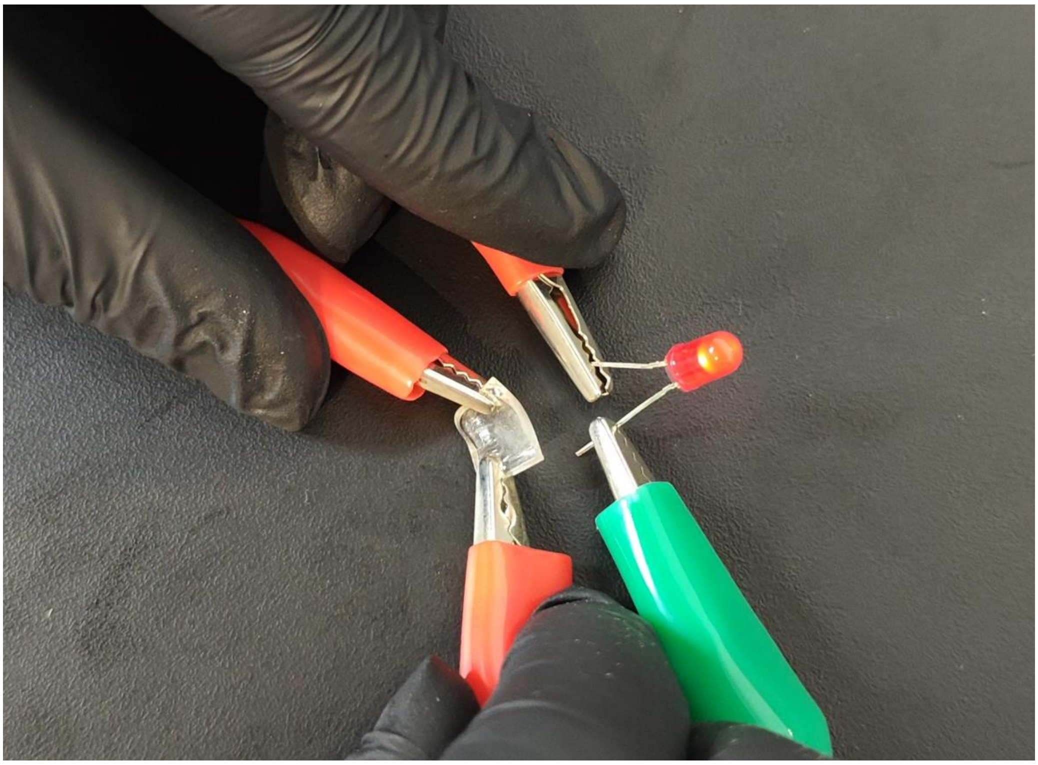

Figure. S7. Connecting HE-LiCl hydrogel at -20 ºC into circuit
